# Molecular structure of human synaptonemal complex protein SYCE1

**DOI:** 10.1007/s00412-018-00688-z

**Published:** 2019-01-03

**Authors:** Orla M. Dunne, Owen R. Davies

**Affiliations:** Institute for Cell and Molecular Biosciences, Faculty of Medical Sciences, Newcastle University, Framlington Place, Newcastle upon Tyne, NE2 4HH UK

**Keywords:** Meiosis, Chromosome structure, Double-strand break, Chiasmata, Synaptonemal complex, Central element, SYCE1, Small-angle X-ray scattering, Biophysics

## Abstract

The reduction in chromosome number during meiosis is essential for the production of haploid germ cells and thereby fertility. To achieve this, homologous chromosomes are first synapsed together by a protein assembly, the synaptonemal complex (SC), which permits genetic exchange by crossing over and the subsequent accurate segregation of homologues. The mammalian SC is formed of a zipper-like array of SYCP1 molecules that bind together homologous chromosomes through self-assembly in the midline that is structurally supported by the central element. The SC central element contains five proteins—SYCE1, SYCE3, SIX6OS1, and SYCE2-TEX12—that permit SYCP1 assembly to extend along the chromosome length to achieve full synapsis. Here, we report the structure of human SYCE1 through solution biophysical methods including multi-angle light scattering and small-angle X-ray scattering. The structural core of SYCE1 is formed by amino acids 25–179, within the N-terminal half of the protein, which mediates SYCE1 dimerization. This α-helical core adopts a curved coiled-coil structure of 20-nm length in which the two chains are arranged in an anti-parallel configuration. This structure is retained within full-length SYCE1, in which long C-termini adopt extended conformations to achieve an elongated molecule of over 50 nm in length. The SYCE1 structure is compatible with it functioning as a physical strut that tethers other components to achieve structural stability of the SC central element.

**Electronic supplementary material:**

The online version of this article (10.1007/s00412-018-00688-z) contains supplementary material, which is available to authorized users.

## Introduction

**Fig. 1:**
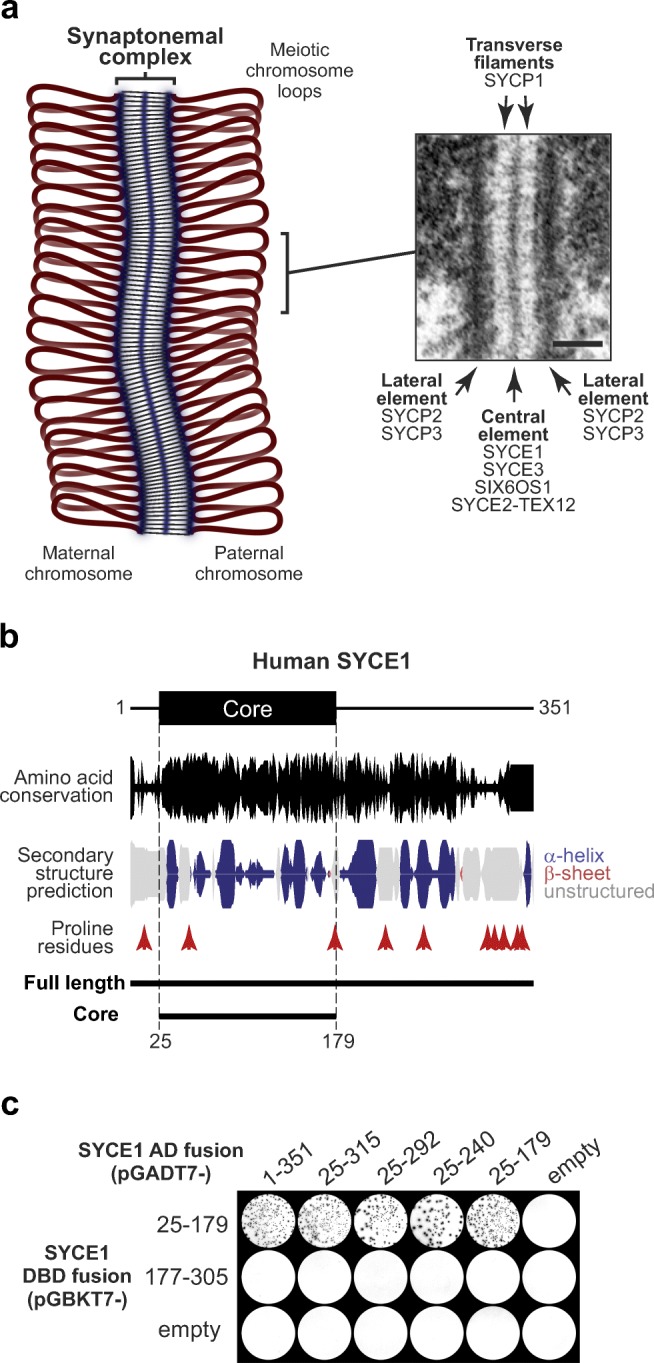
SYCE1 undergoes self-association through amino acids 25–179. **a** Schematic of the synaptonemal complex (SC) binding together a pair of homologous chromosomes in meiosis, with an inset electron micrograph of the SC reproduced from Kouznetsova et al. (2011). Scale bar, 100 nm. The SC has a tripartite structure of two chromosome-bound lateral elements that flank a midline central element and are connected together by a series of interdigitated transverse filaments. SYCE1 is located in the central element alongside SYCE3, SIX6OS1, and SYCE2-TEX12, whereas transverse filaments are formed by SYCP1 and lateral elements contain SYCP2 and SYCP3. **b** Human SYCE1 sequence aligned with its amino acid conservation (calculated as per residue conservation by ConSurf (Celniker et al. 2013) and indicated by peak height) and secondary structure prediction (Drozdetskiy et al. 2015) (α-helix, blue; β-sheet, red; unstructured, grey; with peak height indicating confidence). The location of proline residues and the two principal constructs used in this study (full length, 1–351; core, 25–179) are indicated. **c** Yeast two-hybrid analysis of SYCE1 self-association indicating that amino acid residues 25–179 define the minimum self-associating region. Y187 and Y2HGold yeast strains harbouring pGBKT7 and pGADT7 plasmids (as indicated) were mated and plated onto SD/-Ade/-His/-Leu/-Trp/X-α-Gal. Positive reactions depend on activation of reporter genes *ADE1*, *HIS3*, and *MEL1*. These data are representative of three repeats

The challenging task of generating haploid germ cells by meiosis is achieved through a series of molecular events, coupled with structural and topological changes to chromosomes that result in homologous chromosome segregation following genetic exchange by crossing over (Hunter 2015; Loidl 2016; Zickler and Kleckner 2015). Firstly, chromosomes become organised as linear arrays of chromatin loops such that their external compacted structures reflect the underlying gene sequence linearity (Zickler and Kleckner 2015). Homologous chromosome pairs are then established through inter-homologue recombination searches at sites of induced double-strand breaks (Baudat et al. 2013), a process that is likely supported by rapid prophase chromosomal movements that are driven by cytoskeletal forces transmitted across the nuclear envelope to telomere ends (Stewart and Burke 2014). The local inter-homologue alignments defined by recombination events are then converted into a single global synapsis through assembly of supramolecular protein assembly, the synaptonemal complex (SC) (Fig. 1a) (Cahoon and Hawley 2016). The three-dimensional structure of the SC physically tethers together homologous chromosomes and facilitates recombination intermediate resolution, with the formation of typically one crossover per chromosome arm (Zickler and Kleckner 2015). Crossovers provide the sole physical links between homologues at metaphase I, with their subsequent segregation triggered by arm cohesin cleavage. These intricate molecular processes are essential for meiotic division and fertility in mice (Hopkins et al. 2014; Horn et al. 2013; Kouznetsova et al. 2011; Shibuya et al. 2015), and their defective function has been associated with cases of infertility and miscarriage in humans (Geisinger and Benavente 2016; Handel and Schimenti 2010).

The SC was first identified in crayfish spermatocytes in 1956 (Moses 1956) and has since been identified in almost all meiotic organisms (Westergaard and von Wettstein 1972). It has a classic tripartite structure by electron microscopy, consisting of lateral elements that coat the chromosome axes, a midline 20–40-nm wide central element, and a series of interdigitated transverse filaments that provide the approximately 100-nm separation between lateral elements (Westergaard and von Wettstein 1972) (Fig. 1a). In mammals, transverse filaments are formed by SYCP1 (de Vries et al. 2005; Liu et al. 1996; Schmekel et al. 1996; Schucker et al. 2015), which undergoes self-assembly in the midline and on the chromosome axis to generate a supramolecular lattice that defines the underlying structure of the SC (Dunce et al. 2018). The formation of continuous and functional synapsis by SYCP1 is dependent on its structural support through assembly of midline SC central element proteins SYCE1–3, TEX12, and SIX6OS1 (Bolcun-Filas et al. 2007; Bolcun-Filas et al. 2009; Gomez et al. 2016; Hamer et al. 2008; Schramm et al. 2011), and the chromosome structure imposed by SC lateral element proteins SYCP2–3 and axis proteins (Shin et al. 2010; Syrjanen et al. 2014; Yang et al. 2006; Yuan et al. 2000).

SC central element proteins are proposed to stabilise SYCP1-mediated synapsis and facilitate synaptic extension along the chromosome length by acting as physical struts between SYCP1 molecules (Dunce et al. 2018). Given the three-dimensional nature of the SC, in which there are at least two layers of SYCP1 molecules (Hernandez-Hernandez et al. 2016; Schucker et al. 2015), these physical struts are likely orientated in longitudinal, transverse, and vertical planes. Central element proteins may be divided into two distinct groups. SYCE1, SYCE3, and SIX6OS1 are essential for SC tripartite structure formation so are considered synapsis initiation factors (Bolcun-Filas et al. 2009; Costa et al. 2005; Gomez et al. 2016; Schramm et al. 2011). In contrast, SYCE2 and TEX12 form a constitutive complex that self-assembles into long filaments in vitro and are required for long-range synapsis but not for formation of short stretches of SC tripartite structure in vivo, so are considered synapsis elongation factors (Bolcun-Filas et al. 2007; Davies et al. 2012; Hamer et al. 2006; Hamer et al. 2008). On this basis, it is proposed that SYCE1, SYCE3, and SIX6OS1 stabilise initial synapsis by providing vertical and transverse links between SYCP1 molecules, whilst SYCE2-TEX12 self-assembly provides a longitudinal scaffold for the long-range extension of SYCP1 synapsis along the entire chromosome length (Dunce et al. 2018).

In common with other SC central element components, disruption of SYCE1 leads to complete infertility in mice owing to meiotic arrest in which there is failure of SC formation, with only weak discontinuous loading of SYCP1, and failure of double-strand break repair (Bolcun-Filas et al. 2009). SYCE1 recruitment to the SC is dependent on SYCP1, SYCE3, and SIX6OS1 (Gomez et al. 2016; Hamer et al. 2006; Schramm et al. 2011), but is retained—albeit in short discontinuous stretches with SYCP1—in the absence of SYCE2 or TEX12 (Bolcun-Filas et al. 2007; Hamer et al. 2008). Interactions between SYCE1 and initiation factors SYCE3 and SIX6OS1 have been identified by co-immunoprecipitation and yeast two-hybrid (Gomez et al. 2016; Lu et al. 2014; Schramm et al. 2011). Further, SYCE1 recruitment to the SC is proposed to be mediated by SYCE3 owing to its ability to recruit SYCE1 to SYCP1 cytoplasmic aggregates formed upon heterologous expression in somatic cells (Hernandez-Hernandez et al. 2016). Finally, SYCE1 has been implicated in human infertility, with mutations giving rise to premature truncation or haploinsufficiency having been identified in cases of non-obstructive azoospermia and premature ovarian failure (de Vries et al. 2014; Geisinger and Benavente 2016; McGuire et al. 2011).

Our understanding of mammalian SC protein structure has emerged through crystal structures and biophysical solution studies of SYCP1, SYCE3, SYCE2-TEX12, and SYCP3 (Davies et al. 2012; Dunce et al. 2018; Lu et al. 2014; Syrjanen et al. 2014). In all cases, SC proteins have been found to adopt elongated α-helical “coiled-coil”-like structures, typically in homodimeric or homotetrameric configurations. These rod-like structures define precise lengths whilst permitting lateral or torsional flexibility and thus appear well suited to function as physical struts that define SC geometry. However, we hitherto have no structural information regarding SC central element component SYCE1.

Here, we report the molecular structure of human SYCE1 through multi-angle light scattering and small-angle X-ray scattering solution studies in combination with experimentally directed molecular modelling. The structural core of SYCE1 consists of an α-helical anti-parallel homodimer, formed by its N-terminus, and providing an approximately 20-nm rigid scaffold. SYCE1 C-terminal sequences emanate from this structural core and adopt extended conformations to achieve a flexible SYCE1 molecule of at least 50 nm in length.

## Results

### SYCE1 undergoes self-association through an N-terminal structural core

Biochemical and structural studies of mammalian SC proteins have revealed that they are largely α-helical in nature and typically exist as constitutive homo-oligomers in which α-helical chains are intertwined to form dimeric or tetrameric “coiled-coil”-like structures (Davies et al. 2012; Dunce et al. 2018; Lu et al. 2014; Syrjanen et al. 2014). Sequence analysis revealed α-helical structure prediction for SYCE1 (Fig. 1b), and we have previously identified SYCE1 self-association through yeast two-hybrid (Y2H) analysis (Davies et al. 2012). We therefore hypothesised that SYCE1 forms a similar α-helical homo-oligomeric structure and reasoned that we could identify its structural core through determining its minimum self-associating region by Y2H. We thus performed a Y2H screen using SYCE1 constructs designed on the basis of amino acid conservation and secondary structure prediction (Fig. 1b–c). This revealed a self-association interaction of human SYCE1 N-terminal region of amino acids 25–179 but not of C-terminal region 177–305 (Fig. 1c).

**Fig. 2:**
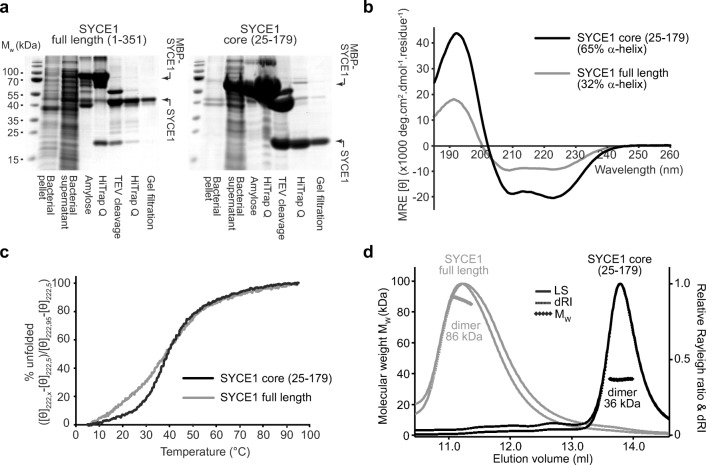
The structural core of SYCE1 is an α-helical homodimer. **a** SDS-PAGE analyses of recombinant expression and purification of human SYCE1 full length (amino acids 1–351) and the human SYCE1 structural core (amino acids 25–179). Recombinant proteins were expressed in *E. coli* and purified through amylose and anion exchange chromatography, followed by TEV cleavage to remove N-terminal MBP tags, with subsequent anion exchange and size-exclusion chromatography. **b** Far-UV circular dichroism (CD) spectra of SYCE1 full length (grey) and SYCE1 core (black) recorded between 260 and 185 nm in mean residue ellipticity, MRE ([θ]) (× 1000 deg·cm^2^·dmol^−1^·residue^−1^). Data were deconvoluted using the CDSSTR algorithm revealing helical content of 32% and 65%, respectively, with normalised r.m.s. deviation values of 0.014 and 0.009. **c** CD thermal detenaturation of SYCE1 full length (grey) and SYCE1 core (black), recording the CD helical signature at 222 nm between 5 and 95 °C, as % unfolded. Melting temperatures were estimated at 38 °C and 39 °C, respectively. **d** Size-exclusion chromatography multi-angle light scattering (SEC-MALS) analysis of SYCE1 full length (grey) and SYCE1 core (black); light scattering (LS) and differential refractive index (dRI) are shown as solid and dashed lines respectively, with fitted molecular weights (Mw) plotted as diamonds across elution peaks. SYCE1 full length and core are dimeric species of molecular weights 86 kDa and 36 kDa, respectively (theoretical dimers—80 kDa and 37 kDa)

In concert with our Y2H studies, we screened for the solubility of human SYCE1 constructs upon recombinant expression in *Escherichia coli*. We were able to purify both full-length SYCE1 (amino acids 1–351) and its N-terminal region (amino acids 25–179; herein referred to as SYCE1 core) through expression with N-terminal MBP-fusion tags that were subsequently removed through enzymatic cleavage during purification (Fig. 2a). In contrast, intermediate C-terminal truncations and further N- and C-terminal truncations beyond amino acids 25–179 proved unstable in solution and prone to non-specific aggregation (data not shown). Thus, we conclude that amino acids 25–179 define the biochemically stable structural core of human SYCE1.

### SYCE1 adopts an α-helical homodimeric structure

Circular dichroism (CD) spectroscopy demonstrated that both full-length and core SYCE1 are largely α-helical in structure owing to characteristic helical spectra in which double-negative peaks are located at 208 nm and 222 nm (Whitmore and Wallace 2008) (Fig. 2b). CD spectra deconvolution estimated α-helical content at 32% and 65%, respectively, indicating that region 25–179 encompasses the majority of α-helical structure within SYCE1 and thus defines its structural core. Structural stability was assessed through thermal denaturation whilst measuring the CD helical signature at 222 nm. This revealed similar apparent melting temperatures for SYCE1 full length and core of 38 °C and 39 °C, respectively (Fig. 2c, Table 1). Further, whilst the melting curve of SYCE1 core demonstrates the classic sigmoidal appearance of cooperative unfolding, full-length SYCE1 demonstrates the presence of additional non-cooperative unfolding events through its initial deviation from a sigmoidal melting curve (Fig. 2c). These findings suggest that structural stability of full-length SYCE1 is conferred by its core region 25–179 (which undergoes cooperative unfolding), with C-termini containing some additional helical structure that is susceptible to thermal denaturation through non-cooperative end-fraying (Fesinmeyer et al. 2005).

**Table 1:** Summary of biophysical data

	SYCE1 core (25–179)	SYCE1 full length (1–351)	MBP-SYCE1	SYCE1-MBP	MBP-SYCE1-MBP	SYCE1 tethered dimer (short)	SYCE1 tethered dimer (long)
Circular dichroism							
α-Helical content (%)	65	32	N/A	N/A	N/A	85	75
Melting temperature (°C)	39.4	38.6	N/A	N/A	N/A	51.2	49.2
SEC-MALS							
Theoretical monomer weight (kDa)	18.6	39.9	63.2	59.8	105.8	37.3	37.8
Experimental molecular weight (kDa)	36.3	86.4	118.1	115.3	189.2	38.7	39.9
Oligomer	Dimer	Dimer	Dimer	Dimer	Dimer	Monomer	Monomer
SEC-SAXS							
*I(0)* (cm^−1^) (from *P(r)*)	0.023 ± 0.0047	0.081 ± 0.0034	0.024 ± 0.0002	0.28 ± 0.0015	0.64 ± 0.0024	0.0052 ± 0.00028	0.02 ± 0.0017
*I(0)* (cm^−1^) (from Guinier analysis)	0.023 ± 0.00006	N/A	0.024 ± 0.00011	0.28 ± 0.0017	0.63 ± 0.0017	0.0051 ± 0.00043	0.02 ± 0.00024
*Rg* (Å) (from *P(r)*)	56.8 ± 1.06	152.1 ± 0.60	77.0 ± 0.34	89.4 ± 0.51	87.2 ± 0.33	60.6 ± 0.32	58.5 ± 0.43
*Rg* (Å) (from Guinier analysis)	56.4 ± 1.66	N/A	76.6 ± 0.66	88.7 ± 5.54	80.1 ± 5.21	67.2 ± 7.53	55.3 ± 5.13
*Rc* (Å)	9.7	N/A	N/A	N/A	N/A	8.9	9.8
*Dmax* (Å)	186	510*	272	309	294	195	183
DAMMIF ab initio model fit (*χ*^2^)	1.07	N/A	N/A	N/A	N/A	1.24	N/A
CORAL rigid-body/random loop parallel model fit (*χ*^*2*^)	1.19	N/A	9.21	15.28	N/A	N/A	N/A
CORAL rigid-body/random loop anti-parallel model fit (*χ*^2^)	1.23	N/A	2.48	2.67	N/A	1.7	N/A
MONSA multi-phase ab initio model fit (*χ*^2^)	1.86	N/A	1.46	1.39	1.52	N/A	N/A

*The *Dmax* may be under-estimated owing to the absence of a Guinier region in this dataset

We utilised size-exclusion chromatography multi-angle light scattering (SEC-MALS) to determine the oligomeric state of SYCE1. This technique couples the chromatographic separation of species on the basis of size and shape with unambiguous mass determination by measuring light scattering at multiple angles alongside differential refractive index, to define the precise molecular species within a protein sample. This revealed that both full-length and core SYCE1 are homodimers, of 86 kDa and 36 kDa, respectively (Fig. 2d, Table 1). Further, despite the instability of intermediate and additionally truncated SYCE1 species, we confirmed their dimeric structure through SEC-MALS analysis of MBP fusions (data not shown). Thus, N-terminal region 25–179 forms the α-helical homodimeric structural core of SYCE1.

### SAXS solution structure of SYCE1 core

**Fig. 3:**
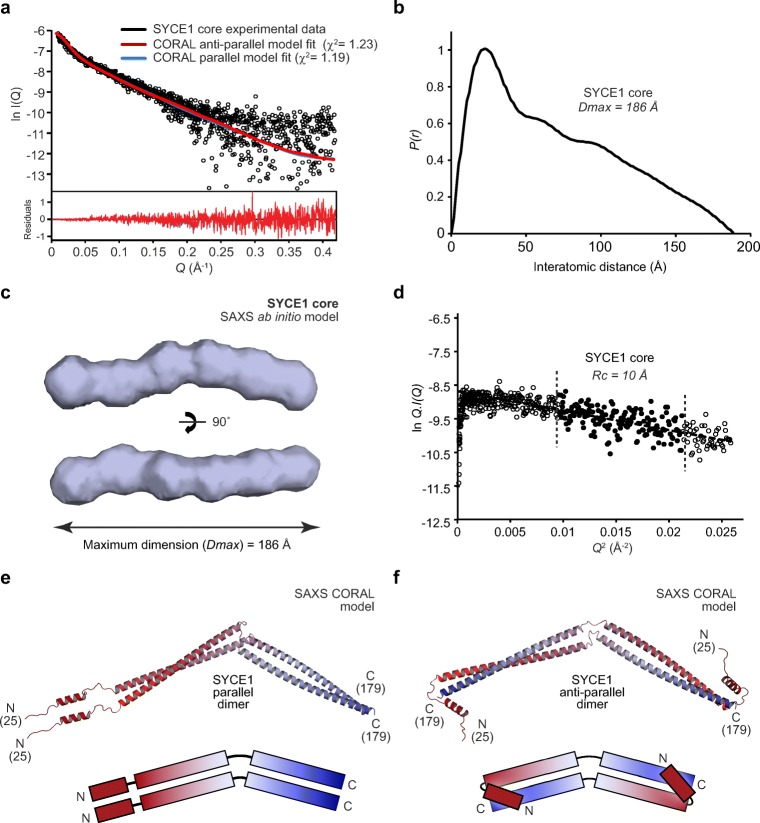
SAXS solution structure of SYCE1 core. **a** Size-exclusion chromatography small-angle X-ray scattering (SEC-SAXS) scattering curve of SYCE1 core (amino acids 25–179), shown in black. Theoretical models of dimeric SYCE1 in parallel and anti-parallel configurations were generated from ideal coiled-coil and helical fragments, through CORAL rigid-body and linker modelling with fitting to experimental SAXS data. Fitted data are shown (parallel, blue; anti-parallel, red) alongside their *χ*^2^ values. Residuals for each fit are shown (inset). **b** SEC-SAXS *P(r)* interatomic distance distribution of SYCE1 core; the maximum dimension (*Dmax*) of the molecule is indicated as 186 Å. **c** SAXS ab initio model of SYCE1 core; an averaged model was generated from 20 independent DAMMIF runs with a NSD value of 0.726 (± 0.058) and reference model *χ*^2^ value of 1.067. **d** SEC-SAXS Guinier analysis to determine the radius of gyration of the cross-section (*Rc*) of SYCE1 core. The linear fit is highlighted in black and is demarcated by dashed lines. The *Q*.*Rc* value was < 1.3 with the *Rc* calculated as 10 Å. **e**–**f** SAXS CORAL models of the SYCE1 dimer, generated through rigid-body and linker modelling with fitting to experimental data, in **e** parallel (*χ*^2^ = 1.23) and **f** anti-parallel (*χ*^2^ = 1.19) configurations, shown alongside schematic illustrations coloured from N- (red) to C-termini (blue)

We elucidated the solution structure of the SYCE1 core through size-exclusion chromatography small-angle X-ray scattering (SEC-SAXS). This technique utilises the X-ray scattering profile of species separated by chromatography to determine the overall size and shape of proteins in solution. The X-ray scattering profile of the SYCE1 core dimer (Fig. 3a) allowed us to determine its radius of gyration through Guinier analysis as 57 Å (Fig. S1a, Table 1) and revealed the elongated nature of the structure through Kratky and Kratky-Debye plots (Fig. S1b–c). SAXS real-space analysis revealed a pair-distance (*P(r)*) distribution function (the distribution of interatomic distances within the protein structure) characteristic of an elongated rod-like structure, with a maximum dimension of 186 Å (Fig. 3b, Fig. S1d, Table 1). We computed ab initio dummy atom models of SYCE1 core based on its *P(r)* distribution, which confirmed its elongated structure and indicated a bend in the middle of the structure, suggesting an overall curved rod-like shape (Fig. 3c). The maximum dimension of SYCE1 core is slightly shorter than the theoretical length of a simple dimeric coiled-coil of 155 amino acids (230 Å). Thus, it cannot be a single continuous α-helical structure but could instead be a coiled-coil of approximately 120 amino acids with at least one helix-loop-helix motif generating a region of coiled-coil containing more than two helices. The SYCE1 sequence contains one proline residue within the 25–179 core region, at amino acid number 51, which we suggest defines a loop within the structural core (Fig. 1b). Importantly, the subsequent sequence could form the approximately 120 amino acid coiled-coil structure determined by the experimental data, with the previous sequence looping back to generate a three or four-helical structure. Accordingly, we determined the SAXS cross-sectional radius as 10 Å (Fig. 3d, Table 1), in keeping with SYCE1 core containing a three or four-helical structure at its maximum width.

We next sought to model the SYCE1 core structure on the basis of SEC-SAXS data. In keeping with the above analysis, we assumed that the structure may include a loop or flexibility surrounding amino acid P51, and reasoned that the dimeric coiled-coil structure could be oriented in either a parallel or anti-parallel manner. We therefore attempted to model a poly-alanine coiled-coil dimer of amino acids 52–179 with a flexible linker to a 15 amino acid poly-alanine α-helix within the 25–50 region (based on helical prediction) and an unstructured N-terminal end. However, we were unable to generate SAXS-directed models of linear coiled-coil structures that provided acceptable fits to the experimental data (data not shown). Instead, we noticed that the SAXS ab initio models consistently included a slight curvature in the middle of the elongated structure, and thus attempted to replicate this in structural modelling by permitting flexibility in the middle of the coiled-coil region. This generated parallel and anti-parallel models in which obtuse angles in the middle of the dimeric coiled-coils provide overall curved rod-like shapes (Fig. 3e–f), which closely match experimental data with *χ*^2^ values of 1.19 and 1.23, respectively (Fig. 3a, Table 1). Thus, parallel and anti-parallel structural models fit to experimental data equally well, requiring the use of other methods to discriminate between these models and determine the helical orientation of the SYCE1 core.

### The SYCE1 structural core is an anti-parallel dimeric coiled-coil

**Fig. 4:**
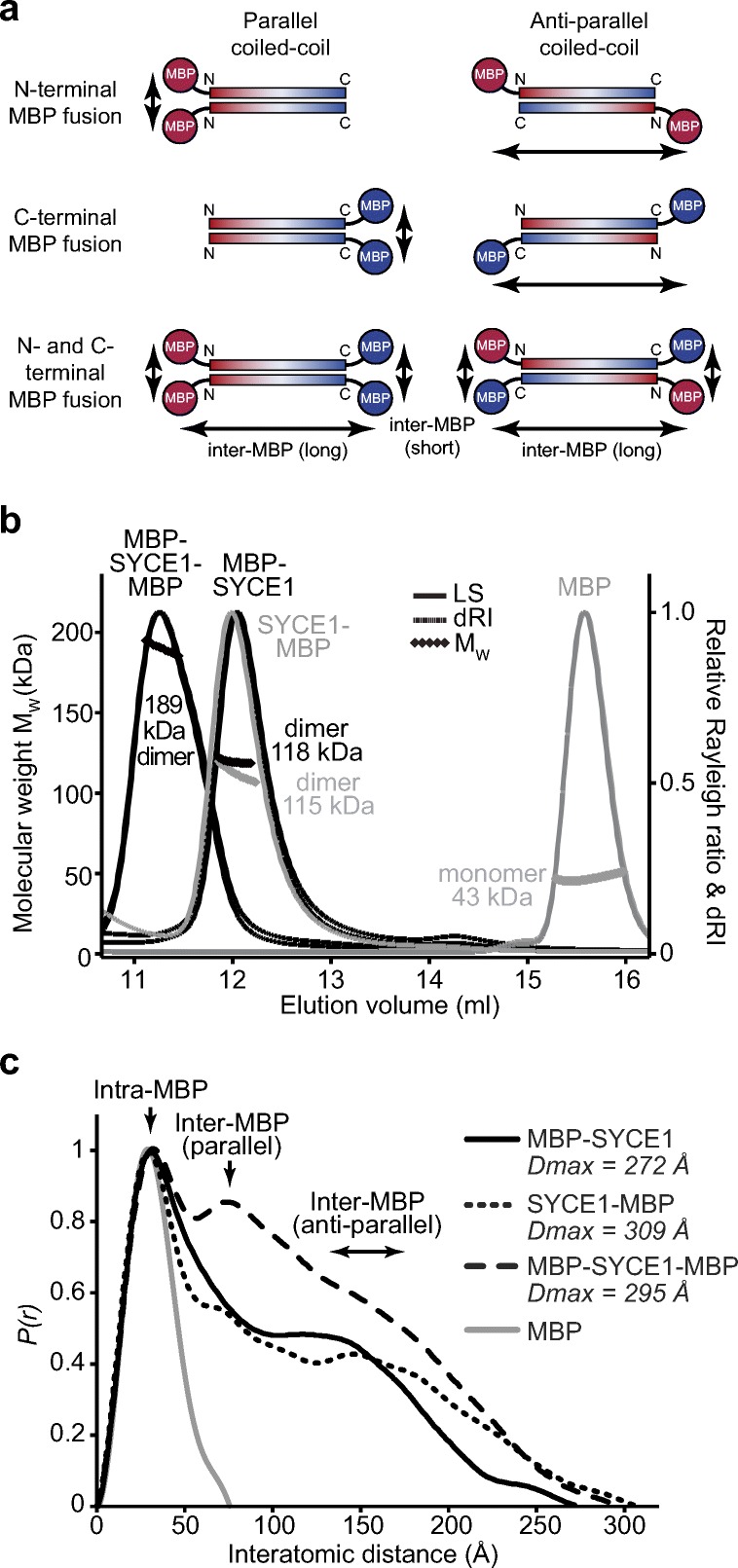
SAXS analysis determining the anti-parallel helical orientation of SYCE1 core. **a** A series of N-, C- and N- and C-terminal fusions of SYCE1 core (amino acids 25–179) were generated. In parallel configurations, individual fusions would demonstrate short inter-MBP distances, whilst anti-parallel configurations would demonstrate long inter-MBP distances. In both cases, the double-fusion would provide long and short inter-MBP distances. **b** SEC-MALS analysis of SYCE1 core fusions demonstrating dimer formation. MBP-SYCE1-MBP (black, left), MBP-SYCE1 (black, right), and SYCE1-MBP (grey, left) showed molecular weights of 189 kDa, 118 kDa, and 115 kDa, respectively (theoretical dimers—211 kDa, 126 kDa, and 118 kDa). The MBP monomer (43 kDa) is shown in grey (right). **c** SEC-SAXS *P(r)* distributions of SYCE1 core fusions demonstrating that SYCE1 is an anti-parallel molecule; MBP-SYCE1 (black, solid), SYCE1-MBP (black, narrow dashes), MBP-SYCE1-MBP (black, wide dashes), and free MBP (grey). Maximum dimensions (*Dmax*) are indicated and the positions of intra-MBP and both parallel and anti-parallel inter-MBP peaks are highlighted

We addressed the question of whether the SYCE1 core coiled-coil dimer adopts a parallel or anti-parallel configuration through SEC-SAXS analysis of SYCE1 core MBP-fusion proteins. This approach exploits the strong scattering of globular proteins in comparison to coiled-coils, and their dominance in scattering curves and *P(r)* distributions, to determine the relative position of MBP molecules and thereby N- and C-terminal orientation within coiled-coil fusion proteins (Dunce et al. 2018). Specifically, MBP molecules fused at either N- or C-termini are related by short interatomic distances in parallel coiled-coils and long interatomic distances in anti-parallel coiled-coils; MBP fusion at both termini provides a positive control in which short and long interatomic distances are present in both parallel and anti-parallel cases (Fig. 4a). We purified N-, C-, and both N- and C-terminal MBP fusions of SYCE1 core and confirmed that they retain its dimeric structure (Fig. 4b, Table 1). We first determined the orientation of globular MBP tags through real-space *P(r)* distribution analysis. The *P(r)* distributions of MBP-SYCE1 and SYCE1-MBP demonstrate the presence of long inter-MBP peaks at 125–175 Å and the lack of short inter-MBP peaks (Fig. 4c and Fig. S2a–b). In contrast, the MBP-SYCE1-MBP double-fusion displayed inter-MBP peaks at both short and long distances of approximately 70 Å and 125–175 Å, respectively (Fig. 4c and Fig. S2a–b, Table 1). Thus, real-space *P(r)* distribution analysis of MBP fusions indicates that SYCE1 core is an anti-parallel coiled-coil.

**Fig. 5:**
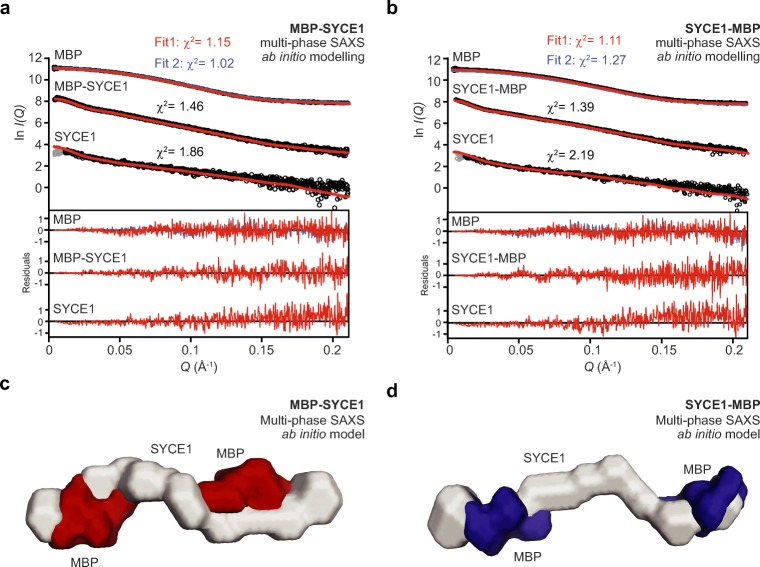
Multi-phase SAXS ab initio modelling of SYCE1 core MBP-fusion proteins. **a**–**d** Multi-phase SAXS ab initio (MONSA) modelling of **a** MBP-SYCE1 and **b** SYCE1-MBP, showing experimental data (black) and ab initio model fits (red and blue); *χ*^2^ values are indicated. Data points shown in grey have been omitted in the Guinier analysis. Fit residuals are shown (inset). **c**–**d** Multi-phase SAXS ab initio (MONSA) models of MBP-SYCE1 and SYCE1-MBP, showing SYCE1 in grey and N- and C-terminal MBP molecules in red and blue, respectively

As a complementary unbiased method, we performed multi-phase SAXS ab initio modelling of SYCE1 core MBP fusions to identify the position of SYCE1 and MBP components within dummy atom reconstructions of fusion proteins. Analysis of MBP-SYCE1 and SYCE1-MBP revealed similar models of an elongated SYCE1 core with MBP molecules located at opposite ends of the structure (Fig. 5a–d), in keeping with an anti-parallel configuration. This is supported by multi-phase SAXS ab initio modelling of the MBP-SYCE1-MBP double-fusion in which N- and C-terminal MBP molecules are orientated at both ends of the molecule (Fig. S2c–e).

**Fig. 6:**
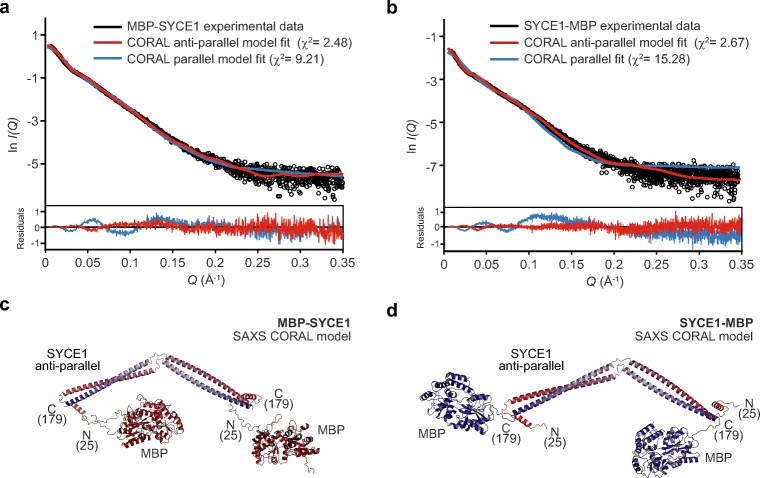
SAXS rigid-body CORAL modelling of SYCE1 core MBP-fusion proteins. **a–b** SEC-SAXS scattering curves of MBP-SYCE1 and SYCE1-MBP. Theoretical models of SYCE1 in parallel and anti-parallel configurations with N- and C-terminal MBP fusions were generated through CORAL rigid-body and linker modelling with fitting to experimental SAXS data. Fitted data are shown (parallel, blue; anti-parallel, red) alongside their *χ*^2^ values; corresponding fit residuals are shown (inset). In both cases, only anti-parallel models are explained by experimental data. **c**–**d** SAXS CORAL models of the SYCE1 anti-parallel dimer with N- and C-terminal MBP fusions, fitted with *χ*^2^ values of 2.48 and 2.67, respectively

Finally, we modelled the SYCE1 core MBP-fusion structures, utilising the refined parallel and anti-parallel SYCE1 core models, through rigid-body and linker modelling against SAXS experimental data. For both N- and C-terminal MBP fusions, anti-parallel models closely fitted experimental data with *χ*^2^ values of 2.48 and 2.67, respectively (Fig. 6a–d, Table 1). In contrast, N- and C-terminal MBP fusions of parallel models showed only poor fits to experimental data, with *χ*^2^ values of 9.21 and 15.28, respectively (Fig. 6a–b, Fig. S2f–g, and Table 1). Thus, through multiple SAXS methods analysing MBP-fusion proteins, we determine that SYCE1 core adopts an anti-parallel dimeric coiled-coil structure.

### The SYCE1 core is stabilised in a tethered dimer construct

**Fig. 7:**
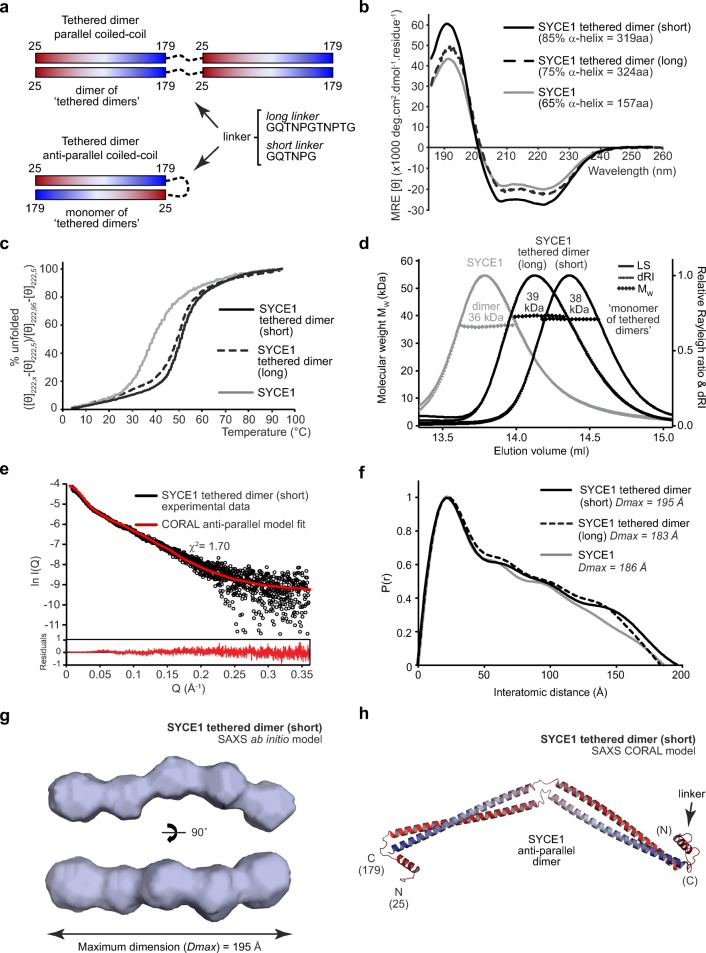
The SYCE1 core anti-parallel structure is stabilised in a tethered dimer construct. **a** A tethered dimer of SYCE1 core (amino acids 25–179) was generated through fusion of two sequences using either a long (GQTNPGTNPTG) or short (GQTNPG) linker. In a parallel coiled-coil configuration, this is predicted to generate a dimer of “tethered dimers” in series, whereas in an anti-parallel configuration, it is predicted to form a monomer of “tethered dimers.” **b** Far-UV circular dichroism (CD) spectra of SYCE1 core tethered dimers with short (black, solid) and long (black, dashed) linkers. Data were deconvoluted using the CDSSTR algorithm revealing helical content of 85% and 75%, respectively, with normalised r.m.s. deviation values of 0.010 and 0.001. SYCE1 core is shown in grey for comparison, exhibiting 65% helicity. **c** CD thermal denaturation of SYCE1 core tethered dimers with short (black, solid) and long (black, dashed) linkers, recording the CD helical signature at 222 nm between 5 and 95 °C, as % unfolded. Melting temperatures were estimated at 51 °C and 49 °C, respectively. SYCE1 core is shown in grey, with a melting temperature of 39 °C. **d** SEC-MALS analysis of SYCE1 core tethered dimers. The short linker and long linker constructs are monomers of “tethered dimers” of 38 kDa and 39 kDa, respectively (theoretical masses—37 kDa and 38 kDa); the SYCE1 core 36 kDa dimer is shown in grey. **e** SEC-SAXS scattering data for the SYCE1 core tethered dimer (short linker) construct, shown in black. A theoretical model of an anti-parallel SYCE1 dimer was generated from ideal coiled-coil and helical fragments, through CORAL rigid-body and linker modelling with fitting to experimental SAXS data (red; *χ*^2^ = 1.48). Corresponding fit residuals are shown (inset). **f** SEC-SAXS *P(r)* distributions of SYCE1 core tethered dimer constructs with short (black, solid) and long (black, dashed) linkers. Maximum dimensions (*Dmax*) are indicated; SYCE1 core is shown in grey. **g** SAXS ab initio model of the SYCE1 core tethered dimer with short linker; an averaged model was generated from 25 independent DAMMIF runs with an NSD value of 0.710 (± 0.043) and reference model *χ*^2^ value of 1.24. **h** SAXS CORAL model of the SYCE1 anti-parallel tethered dimer (short linker), generated through rigid-body and linker fitting to experimental data (*χ*^2^ = 1.70)

To further confirm the orientation of chains within SYCE1 core, we generated a tethered dimer molecule, which would be expected to form a dimer of “tethered dimers” for a parallel orientation and a monomer of “tethered dimers” for an anti-parallel orientation (Fig. 7a). We achieved tethering through long (GQTNPGTNPTG) and short (GQTNPG) linker sequences and in both cases recombinant proteins were highly soluble and stable. We first confirmed α-helical content through CD analysis, revealing a slight increase in helical content for the long linker and further increase for the short linker (Fig. 7b, Table 1). Similarly, CD thermal denaturation analysing helical structure at 222 nm revealed an increase in apparent melting temperature to 49 °C and 51 °C, respectively (Fig. 7c, Table 1). Thus, the SYCE1 core structure is stabilised in the tethered dimer with increased helical content and thermal stability.

We confirmed through SEC-MALS analysis that long and short linker tethered dimers are monomers of “tethered dimers” of 39 kDa and 38 kDa, respectively, indicating an anti-parallel orientation of SYCE1 core chains (Fig. 7d, Table 1). Interestingly, the SEC elution profiles indicate a progressively more compact structure from the SYCE1 core to the long- and short-tethered linkers (Fig. 7d), suggesting structural stabilisation by tethering. SEC-SAXS analysis of SYCE1 core tethered dimers produced *P(r)* distributions and ab initio models comparable to wild type proteins (Fig. 7e–g), with similar radius of gyration and cross-section radius values (Fig. S3b–c, Table 1). Further, the SYCE1 core anti-parallel structural model closely fits to the tethered dimer experimental data, with a *χ*^2^ value of 1.70 (Fig. 7e, h, Table 1). Thus, we conclude that SYCE1 core is an anti-parallel coiled-coil dimer.

What is the structure of full-length SYCE1? SEC-MALS and CD analysis demonstrated that the dimeric structure is retained within full-length SYCE1, with secondary structure and thermal stability accounted for entirely by its 25–179 core (Fig. 2b–d, Table 1). These findings suggest that the additional C-termini of full-length SYCE1 are likely largely unstructured and flexible in solution. In support of this, SEC-SAXS analysis revealed a scattering curve characteristic of an elongated molecule (Fig. S4a), with Kratky-Debye and real-space analysis indicating an extended structure with a maximum dimension of at least 500 Å (Fig. S4b–c, Table 1). Thus, SYCE1 adopts an elongated conformation of a central approximately 20-nm rigid anti-parallel core that is extended through flexible C-termini to achieve a flexible molecule with overall length of at least 50 nm.

## Discussion

The assembly of a structurally and functionally mature SC is dependent on SC central element proteins SYCE1–3, TEX12, and SIX6OS1 (Bolcun-Filas et al. 2007; Bolcun-Filas et al. 2009; Gomez et al. 2016; Hamer et al. 2008; Schramm et al. 2011), which are thought to stabilise the underlying SYCP1 lattice to permit its synaptic extension along the length of meiotic chromosomes (Dunce et al. 2018). Here, we provide the first structural insight into human central element protein SYCE1 through CD, SEC-MALS, and SEC-SAXS solution studies, coupled with SAXS-directed molecular modelling. The SYCE1 structural core, formed by an N-terminal region of amino acids 25–179, is an α-helical dimer that adopts an anti-parallel “coiled-coil”-like structure of approximately 20 nm in length. This anti-parallel dimer structure is retained within full-length SYCE1, with extended C-termini emanating from either side of the rigid core to generate a structure of at least 50 nm in length.

SYCE1 shares common features with other SC proteins in adopting an α-helical homo-oligomeric coiled-coil structure (Davies et al. 2012; Dunce et al. 2018; Lu et al. 2014; Syrjanen et al. 2014). However, it also demonstrates some unique differences from previously described SC structures. Firstly, SAXS data indicate that SYCE1 core undergoes a bending in the middle of its structure to generate an overall curved rod-like shape. We modelled this structure through inserting a hinge in the middle of the dimeric coiled-coil as the simplest means of permitting such flexibility. However, the curved rod-like shape may instead be achieved by a series of short interrupted coiled-coil segments joined together by linker sequences, similar to the coiled-coil configuration observed in the crystal structure of meiotic recombination factor Hop2-Mnd1 (Kang et al. 2015). Indeed, an interrupted coiled-coil structure is supported by the discontinuous pattern of α-helical prediction for SYCE1 core (Fig. 1b) and its CD analysis demonstrating 65% α-helical content. In contrast, previously described SC structures are rigid linear coiled-coils formed of continuous α-helices, with CD analysis determining almost 100% α-helical content (Davies et al. 2012; Dunce et al. 2018; Lu et al. 2014; Syrjanen et al. 2014). Thus, SYCE1 may define a distinct structural class of SC proteins.

What is the role of SYCE1 in SC central element assembly? SYCE1 has been described as a synapsis initiation factor as it is essential for SC tripartite structure formation (Bolcun-Filas et al. 2009). Thus, it is thought to stabilise short stretches of SYCP1-mediated synapsis by providing vertical or transverse links between SYCP1 molecules. This may be achieved through its 20-nm rigid core and/or the wider extended structure provided by its flexible C-termini. The unusual curved rod-like structure of the SYCE1 core could wrap around a linear coiled-coil structure to provide a perpendicular joint between SC components. Such a configuration is likely necessary to join together multiple layers of transverse filaments and to account for the hints of vertical structures observed within the mammalian SC central element (Schmekel et al. 1993), but it has not hitherto been apparent how this could occur given the rigid linear structure of previously reported SC proteins. Thus, SYCE1 may form vertical pillars of the SC that join together transverse filaments bound by other central element proteins to achieve a multi-layered SC.

An important structural or functional role for SYCE1 C-termini is suggested by the identification of mutations associated with human infertility that generate truncated SYCE1 products (de Vries et al. 2014; Geisinger and Benavente 2016; Maor-Sagie et al. 2015). Our biophysical analysis of full-length SYCE1 suggests that C-termini are flexible and favour extended conformations, so they could provide flexible tethers between proteins bound via short peptide motifs. Alternatively, extensive α-helical structure may be induced within SYCE1 C-termini upon protein binding to generate hetero-oligomeric coiled-coils that flank the core anti-parallel homodimer to produce a rigid SYCE1 assembly. This is supported by the presence of predicted α-helical structure within SYCE1 C-termini (Fig. 1b) despite CD analysis demonstrating that the majority of α-helical content within full-length SYCE1 is accounted for by its structural core.

SYCE1 has been reported to interact with synapsis initiation factors SYCE3 and SIX6OS1. An interaction with SYCE3 was detected by co-immunoprecipitation and the ability of SYCE3 to recruit SYCE1 to SYCP1 cytoplasmic aggregates formed upon heterologous expression in somatic cells (Hernandez-Hernandez et al. 2016; Lu et al. 2014; Schramm et al. 2011). Similarly, an interaction with SIX6OS1 was detected by yeast two-hybrid screening and confirmed by co-immunoprecipitation (Gomez et al. 2016). The SYCE1-SIX6OS1 interaction is particularly intriguing as SIX6OS1 shares some common sequence features with SYCE1 so may adopt a similar curved interrupted coiled-coil structure rather than the linear continuous coiled-coil that is typical of other SC proteins. We must now define the precise regions of SYCE1 that mediate its interactions with SYCE3 and SIX6OS1, the structure of their resultant complexes, and the molecular details of how they tether together SYCP1 molecules, in order to understand the full three-dimensional structure of the SC central element.

The methods that we describe for determining the solution structure of SYCE1 overcome the frequent difficulty of obtaining suitable crystals of coiled-coil proteins for crystallographic structure elucidation. Indeed, coiled-coil structures are particularly suitable for structure determination by SAXS as the real-space *P(r)* distribution maximum dimension (*Dmax*) and radius of gyration of the cross-section (*Rc*) define the principal dimensions of a coiled-coil, namely length and width. Further, the SAXS methods we describe for discriminating between parallel and anti-parallel structures provide a simple means for determining coiled-coil helical orientation. In combination with accurate oligomer information from SEC-MALS, SAXS dimensions and orientation may be readily interpreted through known coiled-coil geometry to determine the overall structure of coiled-coil proteins. The roles of coiled-coils as molecular spacers and structural scaffolds mean that they function in a diverse range of cellular functions, most notably in chromosome structure and segregation (Truebestein and Leonard 2016). Thus, we suggest that the methods described herein for coiled-coil solution structure determination may be generally applicable to a wide number of proteins, including those involved in meiotic and mitotic chromosome structure.

## Materials & methods

### Yeast two-hybrid (Y2H)

Constructs of human SYCE1 were cloned into pGBKT7 and pGADT7 vectors (Clontech). Y2H experiments were carried out using the Matchmaker™ Gold system (Clontech) according to the manufacturer’s guidelines. Y187 yeast strain was transformed with pGBKT7 vectors whilst the Y2H gold strain was transformed with pGADT7 vectors. Yeast transformations were carried out using standard lithium acetate methods. Mating of the two strains was carried out in 0.5-ml 2xYPDA at 30 °C, 40 r.p.m., by mixing respective colonies. After 24 h, the cultures were centrifuged and pellets were resuspended in 0.5xYPDA. These were then plated onto SD/-Trp/-Leu to select for mated colonies and onto SD/-Trp/-Leu/-Ade/-His with X-α-gal to detect mated colonies through ADE1, HIS3, and MEL1 reporter gene activation. Plates were then incubated for 5 days at 30 °C.

### Recombinant protein expression and purification

Human SYCE1 sequences were cloned into pMAT11 vectors (Peranen et al. 1996) for bacterial expression as His-MBP fusions with a TEV cleavage site for fusion protein removal. Non-cleavable C-terminal MBP SYCE1 constructs were cloned into pMAT11 with 3XTGS linker sequence. SYCE1 core tethered sequences were cloned with either GQTNPG (short-tethered linker) or GQTNPGGQTNPG (long-tethered linker) from residue 179 to residue 25. SYCE1 constructs were expressed in BL21(DE3) *E. coli* cells (Novagen®), in 2xYT media. Expression was induced with addition of 0.5 mM IPTG with the cells incubated at 25 °C for 16 h. Cells were lysed via sonication in 20 mM Tris pH 8.0, 500 mM KCl, followed by centrifugation. Supernatant was applied to an amylose (NEB) affinity chromatography column, followed by HiTrap Q HP (GE Healthcare) anion exchange chromatography. His-MBP was removed by incubation with TEV protease at 4 °C for 16 h. The cleaved proteins were further purified by HiTrap Q HP (GE Healthcare) anion exchange chromatography followed by size-exclusion chromatography (HiLoad™ 16/600 Superdex 200, GE Healthcare). The purified SYCE1 constructs were concentrated using Microsep™ Advance 3 kDa (PALL) centrifugal filter units and stored at − 80 °C. Protein samples were analysed for purity using Coomassie-stained SDS-PAGE. Protein molecular weights and extinction coefficients were calculated using ExPASY ProtParam (http://web.expasy.org/protparam/) with protein concentrations determined using a Cary 60 UV spectrophotometer (Agilent).

### Circular dichroism (CD)

Far-UV CD spectra were collected using a Jasco J-810 spectropolarimeter (Institute for Cell and Molecular Biosciences, Newcastle University). Wavelength scans were recorded at 4 °C from 260 to 185 nm at 0.2-nm intervals using a 0.2-mm pathlength quartz cuvette (Hellma). Protein samples were measured at 0.2–0.4 mg/ml in 10 mM Na_2_HPO_4_ pH 7.5, 150 mM NaF. Nine measurements were taken for each sample, averaged, buffer corrected, and converted to mean residue ellipticity ([θ]) (× 1000 deg·cm^2^·dmol^−1^·residue^−1^). Spectral deconvolutions were carried out using the Dichroweb CDSSTR algorithm (http://dichroweb.cryst.bbk.ac.uk). CD thermal melts were recorded at 222 nm between 5 and 95 °C, at intervals of 0.5 °C with a 1 °C per minute ramping rate. Protein samples were measured at 0.1 mg/ml in 20 mM Tris pH 8.0, 150 mM KCl, 2 mM DTT, using a 1-mm pathlength quartz cuvette (Hellma). The data were plotted as % unfolded after conversion to MRE ([θ]_222,x_ − [θ]_222,5_) / ([θ]_222,95_ − [θ]_222,5_). The melting temperature was determined as the temperature at which the proteins are 50% unfolded.

### Size-exclusion chromatography multi-angle light scattering (SEC-MALS)

SEC-MALS analysis of protein samples was carried out at concentrations of 6–20 mg/ml in 20 mM Tris pH 8.0, 150 mM KCl, 2 mM DTT. Samples were loaded onto a Superdex™ 200 Increase 10/300 GL (GE Healthcare) column at 0.5 ml/min using an ÄKTA™ Pure (GE Healthcare) system. The eluate was fed into a DAWN® HELEOS™ II MALS detector (Wyatt Technology), followed by an Optilab® T-rEX™ differential refractometer (Wyatt Technology). SEC-MALS data was collected and analysed using ASTRA® 6 software (Wyatt Technology), using Zimm plot extrapolation with a 0.185 ml/g dn/dc value to determine absolute protein molecular weights.

### Size-exclusion chromatography small-angle X-ray scattering (SEC-SAXS)

SEC-SAXS experiments were carried out on beamline B21 at Diamond Light Source synchrotron facility (Oxfordshire, UK). Protein samples at concentrations 6–20 mg/ml were loaded onto a Superdex™ 200 Increase 10/300 GL size-exclusion chromatography column (GE Healthcare) in 20 mM Tris pH 8.0, 150 mM KCl at 0.5 ml/min using an Agilent 1200 HPLC system. The eluate was fed through the experimental cell, with SAXS data recorded at 12.4 keV, in 3.0-s frames with a detector distance of 4.014 m. ScÅtter 3.0 (http://www.bioisis.net) was used to subtract, average the frames, and carry out the Guinier analysis for the *Rg* and cross-sectional *Rg* (*Rc*). Approximate parameters for real-space analysis were obtained using www.bayesapp.org. Final *P(r)* distributions were fitted using PRIMUS. Ab initio modelling was performed using DAMMIF (Franke and Svergun 2009) imposing P1 symmetry. Thirty independent runs were averaged. Multi-phase SAXS ab initio modelling was performed using MONSA (Svergun 1999); rigid-body and linker modelling were performed using CORAL (Petoukhov et al. 2012) with initial idealised poly-alanine coiled-coil and helical fragments generated by CCBuilder 2.0 (http://coiledcoils.chm.bris.ac.uk/ccbuilder2) (Wood and Woolfson 2018). Models were fitted to experimental data using CRYSOL (Svergun 1995). Fit residuals were calculated as the difference between experimental *I(Q)* and calculated *I(Q)* divided by the experimental error as a function of *Q*.

### Protein sequence and structure analysis

ConSurf (http://consurf.tau.ac.il/) was used to calculate amino acid conservation scores for SYCE1 with JNet (http://www.compbio.dundee.ac.uk/www-jpred/) used for secondary structure prediction. The PyMOL Molecular Graphics System, Version 2.0 Schrödinger, LLC was used to generate images of the SAXS ab initio models and rigid-body models.

## Electronic supplementary material

Fig. S1 **SAXS analysis of SYCE1 core.** (**a**) Guinier analysis to determine the radius of gyration (*Rg*) of SYCE1 core with the linear fit highlighted in black and indicated with dashed lines. The *Q*.*Rg* value was <1.3 and the *Rg* was calculated as 57 Å. (**b**) Normalised Kratky plot of SYCE1 core, illustrating that the protein is elongated in nature. (**c**) Flexibility analysis of SYCE1 core; the plateau observed in the Kratky-Debye plot (*P* = 2.0) indicates an elongated/flexible protein. (**d**) SEC-SAXS scattering curve of SYCE1 core with *P(r)* fit (red). Experimental data are shown as black circles with the region used for *P(r)* determination demarcated by a dashed line. (AI 1417 kb)

Fig. S2 **SAXS analysis of SYCE1 core MBP fusions.** (**a**) SEC-SAXS scattering curve of SYCE1 core MBP fusions MBP-SYCE1, MBP-SYCE1-MBP and SYCE1-MBP with their *P(r)* fits (red). Experimental data are shown as black circles with the regions used for *P(r)* determination demarcated by dashed lines. (**b**) Guinier analysis to determine the radius of gyration (*Rg*) of SYCE1 core MBP fusions with linear fits highlighted in black and indicated with dashed lines. The *Q*.*Rg* values were < 1.3 and *Rg* was calculated as 71 Å, 80 Å and 88 Å, respectively. (**c-e**) Multi-phase SAXS ab initio (MONSA) modelling of MBP-SYCE1-MBP, showing (**c**) experimental data (black) and ab initio model fits (red and blue); χ^2^ values are indicated. Corresponding fit residuals are shown (**d**) alongside the (**e**) ab initio model of SYCE1 (grey) and N- (red) and C-terminal (blue) MBP molecules. (**f-g**) SAXS CORAL models of the SYCE1 parallel dimer with (**f**) N- and (**g**) C-terminal MBP fusions, fitted with χ^2^ values of 9.21 and 15.28, respectively. (AI 7801 kb)

Fig. S3 **SAXS analysis of SYCE1 core tethered dimer constructs.** (**a**) SEC-SAXS scattering curves of SYCE1 core tethered dimers with short and long linkers, with *P(r)* fits (red). Experimental data are shown as black circles with the regions used for *P(r)* determination demarcated by dashed lines. (**b**) Guinier analysis to determine the radius of gyration (*Rg*) of SYCE1 core tethered dimers with linear fits highlighted in black and indicated with dashed lines. The *Q*.*Rg* values were < 1.3 and *Rg* was calculated as 67 Å and 54 Å, respectively. (**c**) Guinier analysis to determine the radius of gyration of the cross-section (*Rc*) of SYCE1 core tethered dimers with linear fits highlighted in black and indicated with dashed lines. The *Q*.*Rc* values were < 1.3 and *Rc* was calculated as 9 Å and 10 Å, respectively. (AI 1576 kb)

Fig. S4 **SAXS analysis of full length SYCE1.** (**a**) SEC-SAXS scattering curve of SYCE1 full-length with *P(r)* fit (red), revealing a profile characteristic of an elongated/rod-like structure. Experimental data are shown as black circles with the region used for *P(r)* determination demarcated by a dashed line. Guinier analysis failed to identify a clear linear region within the low-*Q* data, indicating that the maximum dimension of the molecule is beyond the range that can be accurately measured. (**b**) Flexibility analysis of SYCE1 full length; the plateau observed in the Kratky-Debye plot (*P* = 2.1) indicates an elongated/flexible protein. (**c**) SEC-SAXS *P(r)* distribution of SYCE1 full length, showing a radius of gyration (*Rg*) of 138 Å and maximum dimension (*Dmax*) of 510 Å. Given the lack of sufficient low-*Q* data demonstrated by absence of a Guinier region, the true maximum dimension of the molecule may be longer than the *Dmax* determined from real-space analysis. These SAXS findings are consistent with full-length SYCE1 adopting an extended confirmation of over 500 Å in length. (AI 1303 kb)

## Data Availability

All data are available from the corresponding author upon reasonable request.
